# Spit for Science: launching a longitudinal study of genetic and environmental influences on substance use and emotional health at a large US university

**DOI:** 10.3389/fgene.2014.00047

**Published:** 2014-03-06

**Authors:** Danielle M. Dick, Aashir Nasim, Alexis C. Edwards, Jessica E. Salvatore, Seung B. Cho, Amy Adkins, Jacquelyn Meyers, Jia Yan, Megan Cooke, James Clifford, Neeru Goyal, Lisa Halberstadt, Kimberly Ailstock, Zoe Neale, Jill Opalesky, Linda Hancock, Kristen K. Donovan, Cuie Sun, Brien Riley, Kenneth S. Kendler

**Affiliations:** ^1^Virginia Institute for Psychiatric and Behavioral Genetics, Virginia Commonwealth UniversityRichmond, VA, USA; ^2^Department of Psychiatry, Virginia Commonwealth UniversityRichmond, VA, USA; ^3^Department of Psychology, Virginia Commonwealth UniversityRichmond, VA, USA; ^4^Department of Human and Molecular Genetics, Virginia Commonwealth UniversityRichmond, VA, USA; ^5^Department of African American Studies, Virginia Commonwealth UniversityRichmond, VA, USA; ^6^The Wellness Resource Center, Virginia Commonwealth UniversityRichmond, VA, USA

**Keywords:** genes, environment, alcohol, drugs, health, students

## Abstract

Finding genes involved in complex behavioral outcomes, and understanding the pathways by which they confer risk, is a challenging task, necessitating large samples that are phenotypically well characterized across time. We describe an effort to create a university-wide research project aimed at understanding how genes and environments impact alcohol use and related substance use and mental health outcomes across time in college students. Nearly 70% of the incoming freshman class (*N* = 2715) completed on-line surveys, with 80% of the students from the fall completing spring follow-ups. 98% of eligible participants also gave DNA. The participants closely approximated the university population in terms of gender and racial/ethnic composition. Here we provide initial results on alcohol use outcomes from the first wave of the sample, as well as associated predictor variables. We discuss the potential for this kind of research to advance our understanding of genetic and environment influences on substance use and mental health outcomes.

## Introduction

Understanding how genetic, environmental, and developmental influences impact complex behavioral health outcomes is a challenging task. Large samples are necessary, both to utilize increasingly sophisticated molecular genetic methods (because of the small effect sizes of most risk variants) and because of the heterogeneity and subtlety of many developmental pathways. However, characterizing large numbers of subjects phenotypically, across time, along with the needed assessments of present and past environmental risk factors can be cost-prohibitive. Accordingly, achieving the requisite sample size has often required combining samples collected by different groups, having different characteristics, and using varied assessment measures. This introduces noise into any analysis and often means that only the most basic phenotypes are available for study once data are harmonized.

We propose that working with college populations is one way to address these challenges and obtain large numbers of individuals that can be readily phenotyped and followed longitudinally. Importantly, college students are entering a high risk age range for the onset of many psychiatric and substance use outcomes, which have peak onsets in the late teens and early 20 s. Data from the National Comorbidity Survey Replication sample indicate that three quarters of all lifetime cases of DSM-IV diagnoses start by age 24 (Kessler et al., 2005). Forty percent of cases of alcohol abuse have an age of onset (AO) between 16 and 21, and 40% of alcohol dependence cases manifest between ages 17 and 23. One quarter of generalized anxiety disorder cases have an onset by age 20, and one quarter of cases of major depression have an AO of 19. These findings come from a US population, but are consistent with WHO's World Mental Health data, which indicates that approximately three quarters of lifetime mental health disorders begin by the mid-20′s; many of these become evident between the mid-teen years and the mid-20′s (Kessler et al., 2007).

In addition to clinical level diagnostic problems, many college students engage in a range of high risk behaviors that can have both short and long term health implications. This is particularly true in the area of alcohol use. College students show high rates of binge drinking (White et al., 2002, 2004, 2006; White and Swartzwelder, 2009) and are also more likely to report driving under the influence of alcohol (Hingson et al., 2009). Further, alcohol use among college populations is associated with a number of adverse consequences, including academic problems, unwanted sexual encounters, legal consequences, assault, injury, suicide, and death (Wechsler et al., 2002; Hingson et al., 2009).

The transition to college represents a critical developmental period. Many individuals are leaving their family home for the first time. This transition is associated with a variety of new stressors and changing responsibilities. For many students, this period marks changes in family dynamics as they become increasingly independent (Leondari and Kiosseoglou, 2000). During this period, many students move away from prior social supports and enter into new social environments with novel peer influences. Additionally, the college environment often encompasses increases in academic rigor, financial restraints, and personal responsibility for physical and emotional health (Staats et al., 2007; Hicks and Heastie, 2008). These stressors make entering into college a potentially vulnerable period for substance use and emotional health problems.

The present study, called “Spit for Science,” was born out of an attempt to address many of the challenges delineated above associated with large scale studies of genetic, environmental, and developmental influences by studying a large cohort of individuals as they transitioned through this critical developmental phase. To more fully understand the development of substance use and emotional health outcomes in college students we launched a comprehensive, university-wide study, with broad-based genetic, environmental, and phenotypic assessments. Freshman college students commence participation as they arrive on campus, and we follow them longitudinally across their college years and beyond (planned). Because this study is funded largely by the National Institute on Alcohol Abuse and Alcoholism (NIAAA), there is considerable focus on alcohol outcomes, though we have also included brief broad-based assessments of several other substance use and psychiatric outcomes. In this article, we describe the process for launching such a study, and provide some preliminary results on levels of cooperation as well as rates of substance use and emotional health outcomes from the first wave of data collection of the Spit for Science sample.

## Methods

### Project implementation

Approximately 2 weeks before the freshmen were scheduled to arrive on campus, we mailed information about the study to all incoming freshmen and (separately) their parents. The week before Welcome Week all eligible freshmen (age 18 or older) received an e-mail through their university e-mail account inviting them to participate in the project. Survey reminders were sent weekly for the first 4 weeks and then intermittently for the remaining 6 weeks. Students who turned 18 over the course of data collection were invited to participate shortly after their birthdays. The invitation e-mail contained a link to an on-line survey with questions about personality and behavior, as well as family, friends, and experiences growing up. All students that initiated the survey were first led through a consent process that further explained the study and their participation. We estimated the entire survey would take approximately 15–30 min to complete. [Of note, we considerably shortened the survey in the spring based on focus group feedback that survey completion took closer to an hour and that this was the major deterrent to participation.] Upon completion of the survey, students went to a central site at the university to collect payment ($10 and a free “Spit for Science” t-shirt) and provide a saliva DNA sample (hence the “spit” in Spit for Science) for which they received another $10. They also had the option to take informational materials about the DNA component and return at a later time. Students had the option of participating in the survey portion of the project and not the DNA component. We also scheduled “dorm visits” where the research team was available at various residence halls at pre-specified times to give payments and collect DNA in an effort to minimize subject burden.

For the spring survey of the 2011 entering cohort, we mailed hard copy letters to all eligible students (regardless of whether they participated in the fall) informing them about the upcoming second wave of data collection. We included $2 bills in the mailing as an added incentive to participate. Parallel to the fall, e-mail invitations were sent through university e-mail accounts with a link to the on-line survey. Two surveys were programmed for the spring: one was a follow-up survey sent to individuals who had participated in the fall. The other was a survey sent to individuals who did not participate in the fall, giving them a second chance to become part of the project. Their survey consisted of an abbreviated number of questions asking them to retrospectively report on items from the fall survey, and a reduced number of the spring follow-up items. In this way, their total survey length was comparable to the spring follow-up survey, since survey length was cited as a major deterrent to participation in the fall in student focus groups. We plan to follow all participants with annual surveys each spring throughout the duration of their college years and beyond (as funding permits).

Study data were collected and managed using REDCap electronic data capture tools hosted at Virginia Commonwealth University (Harris et al., 2009). REDCap (Research Electronic Data Capture) is a secure, web-based application designed to support data capture for research studies, providing: (1) an intuitive interface for validated data entry; (2) audit trails for tracking data manipulation and export procedures; (3) automated export procedures for seamless data downloads to common statistical packages; and (4) procedures for importing data from external sources.

### DNA collection and extraction

Four milliliter of saliva was collected from each participant in Oragene collection tubes (DNA Genotek, Kanata, Ontario). DNA was isolated following manufacturer's instructions. Briefly, each 4 ml saliva sample was pre-warmed overnight at 50°C. After incubation, each sample was divided into 4 × 1 ml aliquots in 1.5 ml tubes. Forty ul of manufacturer supplied isolation regent was added to each 1 ml aliquot and the tubes were briefly vortexed, incubated on ice for 5 min and centrifuged at 15,000 rpm for 5 min in a benchtop centrifuge. After centrifugation, the supernatant was transferred into a fresh 15 ml Falcon tube and 4 ml of 100% ethanol were added to each tube (less if the original saliva volume was less than 4 ml). Samples were mixed by inversion 15–20 times, incubated at room temperature for 15–30 min and centrifuged again at 15,000 rpm for 5 min. The supernatant was removed from each tube and the pellet was allowed to air dry at room temperature for at least 2 h. During the first year sampling, dried pellets were resuspended in 1 ml TE (10 mM Tris HCl (pH 8.0)/1 mM EDTA). More recently we have reduced this volume to 500 ul to avoid low DNA concentrations.

Following isolation, samples were quantified by spectrophotometry using a Thermo Nanodrop (Thermo Fisher Scientific, Waltham, MA). Our threshold for considering a sample acceptable was a minimum mass of 20 ug, yielding a minimum concentration of 20 ng/ul in 1000 ml. For samples with yields of 10–19.9 ug, the resulting concentrations were too low (<20 ng/ul) for subsequent experimental needs. Purity of the nucleic acid preparation is generally high for DNA isolated from saliva. Across all samples collected and isolated using Oragene tubes and reagents, the mean (SD) OD_260_/OD_280_ ratio was 1.85 (0.12). These samples were reprecipitated by addition of 0.1 volume of 3 M NaOAc (300 mM final concentration) and two volumes of 100% ethanol, incubation at −80°C for 30 min and centrifugation at 15,000 rpm for 5 min. Reprecipitated samples were resuspended in an appropriate volume of TE to yield a concentration >20 ng/ul based on the total amount of DNA reprecipitated. Individuals whose samples did not yield the minimum 10 ug were asked to provide a second saliva sample.

### Other project-related groundwork and activities

We conducted extensive groundwork before launching the Spit for Science project to develop support, raise awareness, and minimize misunderstanding that could surround a large-scale university-wide study with a genetic component. This included meeting with multiple stakeholders across the university, including administrators at multiple levels (the President, Provost, Vice Presidents, Deans), the registrar's office, faculty, staff, and students. We worked with the public relations and media office, and they ran stories about the project both in university outlets and the local media. We developed a close working relationship with the VCU Institutional Review Board (IRB), due to the unique nature of a large-scale study at a major university with highly time-sensitive data collection. All Spit for Science protocols were approved by the IRB. We worked with the university Wellness Resource Center to develop informational materials about alcohol use on college campuses to mail to parents over the summer prior to matriculation alongside informational materials about the study. We developed informational materials about the study and a study-specific website (www.spit4science.vcu.edu). Included in these materials was information about why the study included a genetic component and how confidentiality was handled in the project. A minority faculty member who was part of the research team led focus groups with African American students, parents, and faculty/staff to solicit feedback about the project, which contributed to the creation of a section of the study website addressing concerns about minority participation in genetic research (most specific to the African American community). We conducted a series of “Spit for Science” sponsored talks, forums, and educational activities, to introduce students to the importance of research at universities, to raise awareness about substance use and emotional health issues on college campuses, and to increase understanding about complex trait genetics.

### Measures

The questionnaire was designed to collect broad-based information about substance use and mental health outcomes, as well as related risk and protective factors. When possible, previously validated scales from the literature and/or from the authors' other projects were used. Table 1 provides an overview of the primary constructs of interest that were included and the scales used to measure them. Additional details on the measures are included below.

**Table 1:** **Overview of measures assessed in Spit for Science**.

**Construct**	**Measure**
Alcohol use	Varied items (see text)
Illicit drug use	Varied items (see text)
Antisocial behavior	SSAGA
Anxiety/depression	SCL-90
Binge eating	EDE-Q
Family history	Varied items (see text)
Alcohol expectancies	B-CEOA
Drinking motives	Drinking motives questionnaire
Reasons for not drinking	Varied items (see text)
Personality	Big five inventory
Religiosity	National comorbidity survey
Parenting styles	Parenting styles inventory
Life events	Life events checklist
Social support	Medical outcomes study
Peer group deviance	Varied items (see text)

SSAGA, Semi-Structured Assessment for the Genetics of Alcoholism; B-CEO-A, Brief Comprehensive Effects of Alcohol; SCL-90, Symptom Checklist -90; EDE-Q, Eating Disorder Examination Questionnaire.

#### Alcohol use and problems

Participants were asked whether or not they had ever had a drink of alcohol excluding small tastes and sips. Those who answered yes were asked further alcohol questions, such as how they would describe their current use (self-attribution of use: “abstainer,” “abstainer – former problem drinker in recovery,” “infrequent drinker,” “light drinker,” “moderate drinker,” “heavy drinker,” and “problem drinker”). Participants were asked on how many days they had one or more drinks in the past 30 days and how many drinks they usually had on days that they drank. Both questions were free response. A frequency/quantity variable was then created by multiplying a participant's score on frequency by their score on quantity. Participants were asked at what age they began to drink regularly, defined as drinking at least once a month for 6 months or more. They were also asked how old they were when they first got drunk, defined as having slurred speech or being unsteady on one's feet. Questions related to symptoms of alcohol dependence were included, as adapted from the Semi-Structured Assessment of the Genetics of Alcoholism (Bucholz et al., 1994). Response options for these questions were “Never,” “1–2 times,” “three or more times,” or “don't know.” An alcohol problem score was created by summing the number of symptoms for which a participant endorsed “three or more times.”

#### Illicit drug use

Participants were asked to indicate whether they had ever used the following five drugs for non-medical use: cannabis, sedatives stimulants, cocaine, or opioids. Non-medical use was defined as use without a doctor's prescription, in greater amounts than prescribed, or for other reasons than recommended by a doctor.

#### Nicotine use

Participants were asked to report on how many cigarettes they had smoked in their lifetime. Participants who had smoked at least one cigarette were asked how many times in the past month they had smoked. Items regarding the frequency of cigar and hookah use in the past 30 days were included. Participants were asked how many days in the past 30 they had used cigars, little cigars, or cigarillos and how many days a hookah had been used.

*Antisocial behavior* was assessed with 11 questions (α = 0.65) assessing conduct disorder symptomatology from the Semi-Structured Assessment for the Genetics of Alcoholism (Bucholz et al., 1994). At the fall assessment, participants were asked to report on how often they engaged in the behaviors while in high school. A sum score was created for individuals with more than half (6) nonmissing responses.

*Anxiety and Depression* were measured using a subset of items from the SCL-90 (Derogatis et al., 1973), a measure used both in clinical practice and research to measure psychological symptoms. This self-report questionnaire utilized a past month timeframe, and consisted of 24 empirically derived items from four of the SCL subscales: depression (11 items), somatization (1 item), anxiety (7 items), and phobic anxiety (5 items). Response options were “not at all,” “a little bit,” “moderately,” “quite a bit,” and “extremely.” The anxiety (α = 0.85) and depression (α = 0.89) subscales used in these analyses were created by averaging the responses for those with non-missing answers for more than half of the anxiety and depression questions respectively.

#### Binge eating

Eating disorder symptoms were screened for using a brief number of items derived from the EDE-Q (Fairburn and Beglin, 1994). Here we examine the item that asked participants to report whether there had been times during the last 4 weeks when they consumed what most people would consider an unusually large amount of food.

#### Family history

Participants were asked whether they thought their biological (1) mothers, (2) fathers, (3) siblings, and (4) aunts, uncles, or grandparents had ever experienced problems with (1) alcohol, (2) other drugs, or (3) depression/anxiety. Questions were asked separately for each relative group and outcome.

*Alcohol expectancies* were assessed using a subset of items from The Brief Comprehensive Effects of Alcohol (Fromme et al., 1993). This scale consists of seven subscales: Sexuality (participant expects to enjoy sex more or be a better lover while under the influence of alcohol), Cognitive and Behavioral Impairment (feelings of dizziness or clumsiness), Risk and Aggressiveness (expectations to be loud, boisterous, or noisy; act aggressively; or take risks), Tension Reduction (expectations to feel peaceful or calm), Liquid Courage (expectations to be brave and daring or courageous), Self-Perceptions (expectations to feel guilty or moody), and Sociability (expectations to be sociable or that talking to others would be easier). Each item was rated on a Likert-type scale from 1 (Disagree) to 4 (Agree), and sum scores were computed for each subscale.

*Drinking motives* were assessed with the Drinking Motivations Questionnaire (Cooper, 1994). This scale is broken into four subscales: Social Drinking (α = 0.88, to improve sociability), Conformity Drinking (α = 0.89, to fit in with a group), Drinking to Cope (α = 0.89, to cheer up or gain confidence), and Drinking to Enhance (α = 0.85, because drinking is fun or gives a high). Each item was rated on a Likert-type scale anchored with 1 (Strongly Agree) and 4 (Strongly Disagree). Items were reverse scored and summed so that higher scores represent greater motivation for the subscale construct.

#### Reasons for not drinking

Questions were included about why individuals abstain from drinking or limit their drinking habits (Sher and Rutledge, 2007). Participants were asked to indicate their reasons for not drinking, such as because of its taste, religious obligations, fear of becoming an alcoholic, fear of getting into trouble, concern with not being able to control oneself while under the influence, etc. Each item was rated on a Likert-type scale such that responses ranged from 1 (Not at all Important) to 3 (Very Important). A sum score was computed for each individual such that higher scores indicated the importance of the given reason to abstain from drinking.

*Personality* was measured using the Big Five Inventory (BFI; John and Srivastava, 1999). The BFI consists of five subscales measuring Extraversion (α = 0.84), Agreeableness (α = 0.76), Conscientiousness (α = 0.79), Neuroticism (α = 0.81), and Openness (α = 0.74).

*Religiosity* was measured using items originally derived from the National Comorbidity Survey, a Gallup poll, and the religiousness scale of Strayhorn and colleagues (Kendler et al., 1997b). The nine-item measure was subjected to an exploratory factor analysis with varimax rotation. We retained a single factor consisting of eight of the nine items (α = 0.87) from which factor scores were calculated and used in the subsequent analyses.

*Parenting style* was assessed using the Parenting Styles Inventory (Steinberg et al., 1992), which asks students about the parent or guardian that they lived with while growing up. It consists of two subscales: Parental Involvement and Autonomy Granting. The parental involvement subscale is comprised of nine items (α = 0.82), which measure how involved the parents are in the child's life. The autonomy granting subscale is made up of 9 items (α = 0.68) that measure how much individuality and freedom the individual was given by their parents. Sum scores were computed for individuals with five or more non-missing values.

*Life events* were assessed using items adapted from the Life Events Checklist (Gray et al., 2004). Participants were asked to report on the occurrence of five different stressful events: natural disasters, physical assaults, sexual assaults, other unwanted or uncomfortable sexual experiences, and transportation accidents. Participants were given the response options of “ever,” “in the past 12 months,” or “never happened to me.” Items were summed to yield a total Stressful Life Events score. In addition, an inventory of 15 additional items reflecting potentially stressful life events experienced in the past 12 months (broken engagement, separation from a loved one or close friend, serious illness or injury, trouble with the police, etc.) was included (Kendler et al., 1998). A sum score was created for each individual based on their endorsement of total exposure to the events.

*Social support* was assessed using items adapted from the Social Support Survey of the RAND Medical Outcomes Study (Hays et al., 1995). This consisted of five subscales, each containing one item: Tangible Support (i.e., support in the event of confinement to bed), Emotional/Information Support (i.e., support when good advice during a crisis is needed), Positive Social Interaction (i.e., availability of someone to get together with for relaxation), Affectionate Support (i.e., availability of someone to love you and make your feel wanted), and an additional item regarding availability of someone to confide in or talk about your problems. Participants were asked to indicate how often someone was available to provide the above-mentioned support, in the past 12 months. Participants were given the response option of “none of the time,” “some of the time,” “most of the time,” “all of the time,” or “I don't know.” A sum score was computed for individuals with more than three non-missing indicators, with lower scores indicating less support.

*Peer group deviance* was measured with 12 items taken from two well-validated measures (Johnston et al., 1982; Tarter and Hegedus, 1991). Items asked respondents to report how many of their friends engaged in certain behaviors such as: selling drugs, smoking cigarettes, skipping school, or cheating on tests. Items were presented on a 5-point Likert-type scale anchored with 1 (None) and 5 (All). Higher scores indicate higher levels of deviance among peers.

### Analyses

We report the basic descriptive statistics for the focal variables (i.e., means and standard deviations for continuous variables and distributions for binary or categorical variables). Pearson correlations were used to assess the intercorrelations between the substance use variables. Chi-square tests or *t*-tests were used to examine differences in alcohol use as a function of family history of problems with alcohol, other drugs, or depression/anxiety. Pearson correlations were used to assess the associations between the alcohol variables, mental health outcomes, and other related risk and protective factors. We also ran a series of chi-squared tests, *t*-tests, and regression to determine whether there were systematic differences in participation in the various components of the project (DNA collection, follow-up). All analyses were run in SPSS version 20 (Armonk, New York).

## Results

### Participation

Across the fall data collection period, invitations to participate were sent to 3623 individuals who were registered freshman who were at least 18 years of age. A total of 2056 individuals (57%) completed the survey; 95% (*N* = 1961) of whom came in to pick up their payment. Survey completion was considerably higher among on-campus freshman (62%) than among off-campus freshmen (36%). In following up on this differential participation, we found a number of differences between participants living on and off campus. Males were slightly more likely to live off campus (15 vs. 12% of females; χ ^2^ = 5.94, *p* = 0.02) and African American students were less likely to live off-campus (8 vs. 14% of white and other ethnicity students; χ ^2^ = 12.69, *p* < 0.01). Additionally, those who lived off campus were more likely to report having a job (43 vs. 23% of on-campus students; χ ^2^ = 48.354, *p* < 0.01), and less likely to report participating in sports activities (χ ^2^ = 8.33, *p* = 0.04) and fraternity/sorority parties or events (χ ^2^ = 26.28, *p* < 0.01). However, no significant differences were observed with respect to participation in school (χ ^2^ = 0.81, *p* = 0.85), community (χ ^2^ = 5.42, *p* = 0.14), and church-related activities (χ ^2^ = 5.74, *p* = 0.13).

Of the individuals who came in to pick up their payment, 97% (*N* = 1884) also participated in giving saliva samples for DNA. We did not observe any differences between individuals who participated in the DNA component and those who did not in terms of sex (χ ^2^ = 0.23, *p* = 0.63); ethnicity of participants (χ ^2^ = 4.27, *p* = 0.12); or other major dimensions of substance use and related psychopathology [e.g., amount of alcohol drunk in the past month (*t*_(1072)_ = −0.15, *p* = 0.89); FTND score (*t*_(256)_ = −0.68, *p* = 0.21), symptoms of anxiety (*t*_(1995)_ = 0.43, *p* = 0.66); symptoms of depression (*t*_(1993)_ = 1.55, *p* = 0.12)].

In the spring, invitations to participate were sent to a total of 3924 students. This represented 1964 invitations to participants who had completed the fall survey (note that a small number of students were no longer at the university; we are just beginning to initiate procedures to follow-up individuals who have left the university). Eighty percentage of those individuals completed the follow-up survey (*N* = 1562), 94% of whom picked up their payment. There were small demographic differences associated with attrition, with males (χ ^2^ = 14.20, *p* < 0.01) and white participants (χ ^2^ = 6.04, *p* = 0.05) being somewhat less likely to complete the spring follow-up. However, importantly, we did not observe any differences in terms of parental alcohol problems [χ ^2^_(1)_ = 0.03, *p* = 0.87]; high school conduct problems [*B*_(2020)_ = 0.04, *p* = 0.80]; extraversion [*B*_(2041)_ = −0.16, *p* = 0.62]; baseline peer deviance [*B*_(2015)_ = −0.75, *p* = 0.08]; alcohol frequency [*B*_(1729)_ = −0.23, *p* = 0.34]; alcohol quantity [*B*_(1690)_ = 0.02, *p* = 0.94]; alcohol problems [*B*_(1980)_ = −0.01, *p* = 0.90]; symptoms of anxiety [*t*_(1995)_ = −0.73, *p* = 0.47]; or symptoms of depression [*t*_(1993)_ = 0.12, *p* = 0.91]. A small number of students who participated in the survey portion in the fall, but did not give DNA at that time, chose to do so in the spring (*N* = 59). Invitations were also sent to 1960 individuals who did not participate in the fall; this group consisted of nonresponders from the fall who were still enrolled at the university (*N* = 1567 who were part of the original 3623 invitations), as well as students who had aged up to be eligible for participation (e.g., they were under 18 years of age during fall data collection), and students who had transferred into the university (*N* = 393). Of these individuals, 34% completed the survey (*N* = 659); 93% of whom picked up their payments. Of those who came in, 92% (*N* = 563) also chose to give DNA.

**Figure 1:**
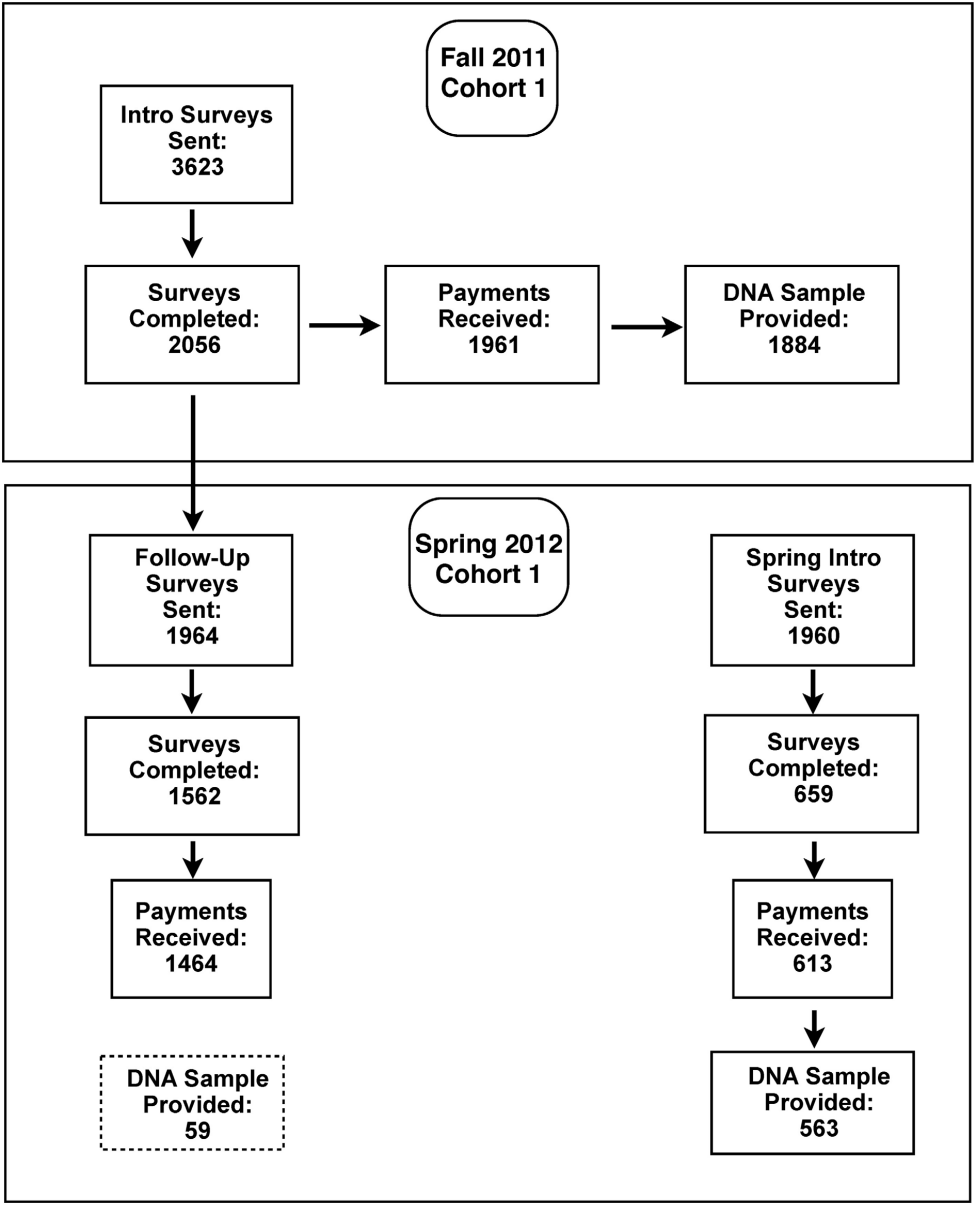
**Overview of participation rates for Cohort 1 across the first year of Spit for Science**.

In summary, over the course of the first year of the project 4016 invitations were sent, with an overall participation rate of 68% (*N* = 2715); 95% of whom picked up their payments. Of the 2574 participants who picked up their survey payments, 97% also chose to participate in the DNA part of the study (*N* = 2506). Each e-mail invitation contained the option to withdraw from the study by clicking on a link. Only 17 students (<1%) withdrew from the project. Figure 1 provides an overview of participation rates.

### DNA sampling results

In the first cohort, a total of 2506 saliva samples were collected and processed. The range of DNA yields and proportion of samples in each bin are shown in Table 2. 2255 samples (90.0%) met or exceeded the threshold of 20 ug total DNA. A further 150 (6.0%) samples with total DNA yield in the range 10–19.9 ug were reprecipitated. A total of 101 individuals (4%) whose samples yielded <10 ug were referred for resampling and 91 were successfully recontacted (with the remainder having separated from the University). Of 91 recontacts, 49 (53.8%) were resampled, and 40 (44.0%) gave total yields at or above the threshold value. DNA purity from this protocol is generally very good, with median OD_260_/OD_280_ ratios of 1.86; 75% of samples have an OD_260_/OD_280_ ratio ≥ 1.8 and 90% of samples have an OD_260_/OD_280_ ratio ≥ 1.7.

**Table 2:** **DNA yields obtained from *N* = 2506 4 ml saliva sample isolations from first year cohort**.

**DNA yield**	***N***	**%**
≥100 ug	991	39.5
50–99.9 ug	682	27.2
20–49.9 ug	582	23.2
10–19.9 ug	150	6.0
<10 ug	101	4.0
Total	2506	100

### Demographics

The gender breakdown for the Spit for Science participants was 60% female, 40% male, compared to the overall fall 2011 freshman class breakdown of 57% female, 43% male. The racial distribution of the Spit for Science sample (with the breakdown for the university shown in parentheses) was 15% (14%) Asian, 20% (19%) African American, 7% (8%) Hispanic, 6% (5%) more than 1 race, and 50% (48%) White. Accordingly, the sample of participants closely maps onto overall VCU demographics in terms of gender and racial/ethnic distribution.

### Descriptives from fall data

The number of students who reported using alcohol was 1449 (70.5%). Of the participants who drank, most students self-described as infrequent drinkers (38.0%), with 22.1% describing themselves as light drinkers, 22.3% as moderate drinkers, 14.0% as abstainers, and 3.7% as heavy or problem drinkers. Students reported drinking a mean of 3.6 days in the past 30 days (*SD* = 4.8), with an average of 3.9 (*SD* = 2.7) drinks consumed per drinking occasion. The mean age of regular drinking was 16.4 years (*SD* = 1.3), and the mean age of first getting drunk was 16.1 years (*SD* = 1.6). The vast majority of students reported no alcohol dependence symptoms (78.0%), 297 (14.4%) reported 1 symptom, 90 (4.4%) reported 2, and 65 (3.1%) reported 3 or more. As expected, smoking cigarettes was the most common form of tobacco use, with 745 students (36.2%) reporting ever having smoked a cigarette. However, 421 students (20.5%) also reported having smoked cigars in the past month, and 548 (26.7%) reported hookah use in the past month. With respect to other drug use, 837 (40.7%) of students reported ever using cannabis, 132 (6.4%) reported ever using sedatives, 217 (10.6%) reported ever using stimulants, 77 (3.7%) reported ever using cocaine, and 93 (4.5%) reported ever using opioids. Table 3 shows the intercorrelations between the substance use variables. Ever use of alcohol was most highly correlated with ever use of cannabis and cigarettes. Higher frequency/quantity of alcohol use was associated with increased likelihood of using other substances. Ever use of cigarettes was more highly correlated with use of illicit drugs other than cannabis than ever use of alcohol was at this age. Use of cigarettes or cannabis were about equally correlated with use of other illicit drugs. Additionally, use of any of the other illicit drugs was most strongly correlated with use of other forms of illicit drugs.

**Table 3:** **Inter-correlations between substance use variables**.

	**1**	**2**	**3**	**4**	**5**	**6**	**7**	**8**	**9**
Ever use—alcohol	1								
Alcohol frequency/quantity	0.31^**^	1							
AD Sx	0.24^**^	0.57^**^	1						
Ever use—cigarettes	0.39^**^	0.34^**^	0.31^**^	1					
Ever use—cannabis	0.45^**^	0.39^**^	0.33^**^	0.53^**^	1				
Ever use—sedatives	0.15^**^	0.29^**^	0.33^**^	0.26^**^	0.29^**^	1			
Ever use—stimulants	0.19^**^	0.30^**^	0.29^**^	0.36^**^	0.38^**^	0.53^**^	1		
Ever use—cocaine	0.10^**^	0.25^**^	0.25^**^	0.21^**^	0.21^**^	0.38^**^	0.45^**^	1	
Ever use—opioids	0.11^**^	0.18^**^	0.21^**^	0.20^**^	0.22^**^	0.52^**^	0.45^**^	0.38^**^	1

AD Sx, Alcohol Dependence Symptom Count.

** p ≤ 0.01.

Table 4 shows the number of students who reported a perceived family history of relatives having a drinking problem, a problem with other drugs, or problems with depression or anxiety, as well as differences in rates of alcohol use and problems as a function of family history. Students who reported a family history (as indexed by any relative type) of alcohol or other drug problems, or problems with depression/anxiety, all showed elevated rates of having initiated alcohol use. Having a mother with perceived alcohol problems was also associated with a significantly higher frequency/quantity of alcohol use, although trends were in the expected direction (with higher rates of use) among those with any positive family history, despite the results not reaching statistical significance. In addition, a positive family history of any kind was associated with significantly increased rates of alcohol dependence symptoms.

**Table 4:** **Endorsement rates for perceived family history of substance use and other mental health problems, and association with alcohol use outcomes**.

		**Prevalence**		**Have used alcohol**		**Frequency/quantity**		**Alcohol dependence Sx**	
	**Family history**	***N***	**%**	**Yes (%)**	**No (%)**	**Yes**	**No**	**Yes**	**No**
Alcohol	Mother	175	8.8	81.9^**^	70.6	21.86^*^	14.56	0.89^**^	0.54
	Father	432	21.8	79.6^**^	68.4	17.85	14.94	0.68	0.55
	Either parent	533	25.9	80.0^**^	67.3	18.65^*^	14.22	0.74^**^	0.51
	Extended family	1093	54.8	78.7^**^	62.3	16.64	13.87	0.66^**^	0.49
	Sibling	205	10.3	83.2^**^	69.9	19.17	15.01	0.71	0.56
Other drugs	Mother	121	6.1	85.0^**^	70.6	20.89	14.96	0.93^**^	0.56
	Father	288	14.4	79.4^**^	69.5	18.28	15.66	0.77^*^	0.57
	Either parent	352	17.1	80.0^**^	68.7	17.31	15.45	0.78^**^	0.55
	Extended family	615	30.8	81.4^**^	65.9	15.31	16.28	0.74^**^	0.53
	Sibling	226	12.6	83.0^**^	69.7	19.36	14.95	0.71	0.55
Depression/anxiety	Mother	780	39.2	78.5^**^	67.5	16.07	15.37	0.66^*^	0.53
	Father	493	24.8	78.9^**^	68.1	15.89	15.51	0.67^*^	0.53
	Either parent	944	45.9	78.2^**^	64.6	16.00	15.83	1.04^**^	0.88
	Extended family	919	46.2	78.7^**^	65.1	16.49	14.69	0.69^**^	0.47
	Sibling	501	28.0	77.2^**^	69.3	17.22	14.78	0.73^**^	0.49

* p < 0.05.

** p < 0.01.

Table 5 shows the means and standard deviations of the other variables and their correlation with the alcohol use variables. Most of the variables included as potential risk/protective factors showed the expected correlations with alcohol use outcomes. Correlations were generally modest, in the range of 0.05–0.25. The variables most strongly correlated with having ever used alcohol were high school peer group deviance (*r* = 0.39) and the alcohol expectancy self-perceptions subscale (expecting to feel guilty or moody if one drinks; inversely associated, *r* = −0.38). Two other alcohol expectancy subscales also showed correlations approaching 0.30 with ever use of alcohol: tension reduction (*r* = 0.28) and sociability (*r* = 0.29). The variables most strongly correlated with frequency/quantity of alcohol use were the drinking motives subscales involving drinking for social reasons (*r* = 0.34) and drinking because it's fun/to get a high (*r* = 0.37), antisocial behavior (*r* = 0.31), and high school peer group deviance (*r* = 0.38). Alcohol dependence symptoms were most strongly associated with peer group deviance (*r* = 0.37) and antisocial behavior (*r* = 0.30). The associations with the drinking motive subscales drinking for enhancement effects (*r* = 0.26) and drinking for social reasons (*r* = 0.22) were also notable.

**Table 5:** **Means, standard deviations, and correlations with alcohol variables**.

	***N*^∧^**	**Range**	**Mean**	***SD***	**Ever use**	**Freq/quant**	**AD Sx**
**DRINKING MOTIVES**							
Social drinking	1461	5–20	14.33	3.97	0.06^*^	0.34^**^	0.22^**^
Conformity drinking	1463	5–20	7.30	3.38	−0.09^**^	−0.05	0.01
Drinking to cope	1462	5–20	9.78	4.29	0.01	0.18^**^	0.24^**^
Drinking to enhance	1462	5–20	13.34	3.88	0.07^**^	0.37^**^	0.26^**^
Reasons for not drinking	1463	1–72	37.32	12.19	−0.006	−0.14^**^
**ALCOHOL EXPECTANCIES**							
Sexuality	1785	2–8	4.20	1.98	0.20^**^	0.23^**^	0.19^**^
Cognitive and behavioral impairment	1979	2–8	6.81	1.47	−0.12^**^	−0.09^**^	−0.04
Risk and aggressiveness	1934	3–12	7.93	2.32	−0.15^**^	0.09^**^	0.11^**^
Tension reduction	1939	2–8	4.96	1.86	0.28^**^	0.14^**^	0.05^*^
Liquid courage	1951	2–8	5.73	1.89	0.10^**^	0.18^**^	0.16^**^
Self-perceptions	1950	2–8	4.46	1.94	−0.38^**^	−0.20^**^	−0.10^**^
Sociability	1956	2–8	6.65	1.63	0.29^**^	0.24^**^	0.18^**^
Religiosity	1849	−1.9–1.9	0.00	1.00	−0.16^**^	−0.09^**^	−0.06^*^
**PARENTING STYLES**							
Parental involvement	2005	9–36	29.25	5.08	0.05^*^	0.04	−0.02
Autonomy granting	2000	9–34	22.02	4.68	−0.02	−0.02	−0.01
Antisocial behavior	2022	11–39.6	15.53	3.49	0.26^*^	0.31^**^	0.30^**^
Anxiety	1999	1–4.86	1.67	0.70	0.02	−0.01	0.14^**^
Depression	1997	1–5	1.91	0.76	0.04^*^	−0.02	0.12^**^
Binge eating	1961	0–1	0.20	0.40	0.06^**^	0.06^*^	0.08^**^
Stressful life events	2010	0–5	1.78	1.21	0.09^**^	0.07^**^	0.15^**^
Life event inventory	2017	0–9	1.45	1.49	0.17^**^	0.13^**^	0.13^**^
**PERSONALITY**							
Extraversion	2043	8–40	27.10	6.22	0.15^**^	0.20^**^	0.14^**^
Agreeableness	2043	14–45	35.00	5.70	−0.01	−0.06^*^	−0.07^**^
Conscientiousness	2043	12–45	31.51	5.71	−0.06^*^	−0.11^**^	−0.11^**^
Neuroticism	2043	8–40	23.06	6.23	0.05^*^	−0.04	0.05^*^
Openness	2043	18–50	38.23	5.76	0.11^**^	0.05^*^	0.05^*^
Social support	1965	5–20	16.23	3.47	0.06^*^	0.09^**^	−0.004
Peer group deviance	2017	12–55	24.00	8.09	0.39^**^	0.38^**^	0.37^**^

Abbreviations: SD, standard deviation; Freq/Quant, Frequency/Quantity; AD Sx, Alcohol Dependence Symptoms.

∧ Ns vary due to certain questions only being asked of subsets of the sample (e.g., drinking motives only being asked to individuals who reported alcohol use), as well as some missing data.

* p ≤ 0.05.

** p ≤ 0.01.

## Discussion

In this paper we have described methods and initial results from a large, genetically informative study of undergraduate students at a major research university. With considerable effort put into designing and raising awareness about the study, we were able to obtain both phenotypic and genotypic data from a large sample that closely represents the diverse, urban university target population. Nearly 70% of eligible participants enrolled in the project by completing a survey during their first year at the university, and nearly all of the individuals who participated in the survey component were also willing to provide a DNA sample (98%). We believe these results provide strong support for the feasibility of launching large-scale genetically-informative projects at universities. Based on the response rate achieved in the first cohort, we project an eventual sample size of *N* = 7500 with current funding over our three planned cohorts. The DNA samples are currently being genotyped on the Affymetrix Biobank Version 2 Array, which contains both rare variation (exome and structural variation), as well as an imputation GWAS grid. These data will be informative for gene identification for quantitative alcohol use and mental health outcomes (and other behavioral traits that were collected, such as personality), and can be combined with other samples to contribute to meta-analyses. In addition, polygenic risk scores can be created in the sample based on results from meta-analyses for a variety of behavioral health outcomes to study how these genetic risk scores impact outcomes in the college students, and interact with other environmental and social factors. The longitudinal nature of the data collection will allow us to study how genetic and environmental influences impact trajectories of substance use and mental health across time. In addition, students were informed that the data they provided could be used to select students to invite them to participate in future spin-off projects. In this way, we have created a large database from which individuals can be selected either based on phenotypic or genotypic data for more intensive studies. For example, we plan to invite subsets of the students to participate in more intensive lab-based protocols, to include neuroimaging at our university brain imaging facility.

Our response rate should be viewed in the context of similar prior US studies. A 2000 meta-analysis identified 68 US web surveys of college populations with a mean response rate of 39.6% (Cook et al., 2000). Similar studies published since report a range of cooperation rates including 21.5% in 2000–2001 (Sax et al., 2003), 27.9% in 2005 (Jans and Roman, 2007) and especially 2011 data from the National Survey of Student Engagement (Nsse, 2011) which, with 636 colleges and universities used a web-survey, had an overall response rate of 34%. We were able to identify only one study with a response rate superior to that obtained in this study (79.5%); however, that project focused only on a target group of on-campus freshman. Thus, our total response rate of 57% at our first wave and ~70% overall compares quite favorably with those obtained in other similar efforts.

Importantly, rates of substance use in the Spit for Science project map onto other large studies of college-age populations. We assessed the representativeness of our sample in terms of substance use by comparing rates of substance use for the fall data collection in the Spit for Science sample with rates of substance use reported in Monitoring the Future (MTF) in their post 12th grade assessment (Johnston et al., 2010). Rates of substance use are very similar between the samples: in Spit for Science 72% of participants reporting having tried alcohol, compared to 71% in MTF. The prevalence of having tried cigarettes was 38% in Spit for Science and 42% in MTF; marijuana: 41% in Spit for Science and 44% in MTF; stimulants: 11% in both samples; sedatives: 6% in Spit for Science and 8% in MTF, and cocaine: 4% in Spit for Science and 5% in MTF. Data from another large survey of college students, The College Alcohol Study (Knight et al., 2002), conducted by the Harvard School of Public Health with data on 23,751 students from 119 4 years colleges across the United States also suggest very similar rates of alcohol problems: 13% of Spit for Science participants endorsed two or more alcohol dependence symptoms compared to 14% of students in the College Alcohol Study.

Further, reports of parental history of substance use and psychiatric problems are in line with larger epidemiological samples (e.g., NESARC, NCS). For example, 26% of VCU students reported that their mother or father ever had a drinking problem, comparable to the 34% of the NESARC participants and 31% of the NCS participants who reported a parental history of alcohol problems (Cuijpers and Smit, 2001; Thompson et al., 2008). Similarly, 17% of our students reported a parental drug history compared to 16% of NESARC participants who reported a parental history of problems with drugs (Elbogen and Johnson, 2009). While 46% of VCU students reported a parental history of anxiety/depression (categories were combined in our study), 32% of NESARC participants reported a parental history of problems with depression (Lizardi et al., 2009), and 14.8% of NCS participants reported a family history of problems with anxiety (Kendler et al., 1997a).

Several of the initial findings of predictors of alcohol use outcomes in the sample are of note. It is interesting that the two alcohol expectancy subscales that were among the strongest predictors of alcohol use represent different reasons for drinking: drinking to reduce tension and drinking to enhance sociability. It suggests that this heterogeneity exists in the college population and that understanding these different pathways to alcohol outcomes in college students is an important area for further exploration. We also found that among the drinking motives, drinking to cope, and social drinking were both positively correlated with alcohol frequency/quantity and alcohol dependence symptoms. The importance of social and interpersonal motivations for college student alcohol use is found throughout the significant predictors (e.g., extraversion is the personality dimension most strongly associated with alcohol outcomes), which has implications for messaging aimed at reducing risky alcohol use among college students. The fact that peer group deviance is among the strongest predictors of alcohol use, quantity/frequency of use, and alcohol dependence symptoms underscores the importance of peer influences at this developmental stage. The parenting variables measured here showed essentially no correlation with alcohol use outcomes at this age. The (unexpected) positive, albeit weak, association between social support and alcohol outcomes at this age could also reflect the association between friends and alcohol use at this developmental stage, as the social support items reflect interpersonal relationships. In short, these findings suggest there may be multiple pathways to problematic alcohol use in college populations, as has been found in other groups (Zucker, 2006, 2008; Hussong et al., 2011). Understanding these different pathways to alcohol outcomes in college students is an important area for further exploration.

Of the other dimensions of mental health that were measured, antisocial behavior was most strongly associated with the alcohol outcomes. This is to be expected based on the robust literature associating these variables (Moss and Lynch, 2001; Armstrong and Costello, 2002). It is interesting that anxiety and depression scores were both more strongly associated with alcohol dependence symptoms than with patterns of alcohol use (initiation, frequency/quantity). This may suggest that these students are an important group to target for intervention efforts.

Studies of this kind provide a unique opportunity to uncover the genetic and environmental underpinnings of behavioral health outcomes. Smaller studies of college students have formed the foundation of much of the evidence base of the psychological literature. In recent years, there has been a growing interest in genetics by psychologists and other social scientists, as it has become widely recognized that most human behavior is influenced by both genetic and environmental factors. Most of these studies have had small sample sizes and are likely underpowered to examine genetic effects (Duncan and Keller, 2011). Expanding the way we think about working with college students to develop more wide-reaching, larger studies may be the next step in taking college research to new levels. With increasingly large samples needed for genetic studies, working with college populations may provide an opportunity to considerably advance our understanding of how genetic and environmental influences impact behavioral health. If our efforts could be duplicated and expanded at other universities to create a series of projects of this sort, it could hold considerable promise for obtaining the very large numbers of subjects necessary to critically advance our understanding of genetic pathways.

Studying college populations also has a number of limitations. College students are a selected population, which may limit the generalizability of certain findings. We believe that this will be less of an issue with respect to gene identification, as previous studies from the genetic epidemiology literature of substance use indicate that genetic influences are stronger in environments that promote alcohol use (Dick and Kendler, 2012), and the and the college setting has been demonstrated to be one such environment (Timberlake et al., 2007). However, findings about gene-environment-development pathways may be less generalizable beyond college populations. College students are exposed to some unique factors that may impact their alcohol use, such as Greek system involvement (Verges and Sher, 2012). However, these college-specific contextual risk and protective factors may still be informative about general etiological mechanisms. Another consideration is that on average, college students come from higher socio-economic backgrounds than individuals who do not go on to college (Lenk et al., 2012). That said, there has been a dramatic shift in the demographics of college student populations, with far more first generation, lower income, and minority students now attending college (Gallagher, 2012). These demographics vary dramatically across colleges. According, the representativeness of college samples will vary. Conclusions drawn from college populations should be viewed within the context of that particular sample and the question under investigation.

Launching a university-wide research effort also provides an impetus for bringing together faculty from around the campus with diverse, complementary interests, and for creating unique academic experiences for the students. Research on a university-wide scale provides visibility to showcase the importance of research at universities to incoming students who may be less aware of universities' research missions and more narrowly focused on their classroom learning opportunities when they first start college. It also provides a platform for initiating discussions about important health-related issues among college students, including how genetic and environmental factors contribute to health-related outcomes, and, in the case of our project, initiating discussions about substance use and emotional health on college campuses. Accordingly, although considerable resources are necessary to organize such an effort, it can be a win-win-win situation for the researcher, the students, and the university.

### Conflict of interest statement

The authors declare that the research was conducted in the absence of any commercial or financial relationships that could be construed as a potential conflict of interest.
